# A Field Study of Nonintrusive Load Monitoring Devices and Implications for Load Disaggregation

**DOI:** 10.3390/s23198253

**Published:** 2023-10-05

**Authors:** Ebony Mayhorn, Joshua Butzbaugh, Alan Meier

**Affiliations:** 1 Pacific Northwest National Laboratory, Richland, WA 99354, USA; joshua.butzbaugh@pnnl.gov; 2 Lawrence Berkeley National Laboratory, Berkeley, CA 94720, USA; akmeier@lbl.gov

**Keywords:** nonintrusive load monitoring, field evaluation, load disaggregation, accuracy, detection, interoperability, residential buildings, commercial buildings, appliances, plug loads, energy monitoring

## Abstract

Evaluations of nonintrusive load monitoring (NILM) algorithms and technologies have mostly occurred in constrained, artificial environments. However, few field evaluations of NILM products have taken place in actual buildings under normal operating conditions. This paper describes a field evaluation of a state-of-the-art NILM product, tested in eight homes. The match rate metric—a technique recommended by a technical advisory group—was used to measure the NILM’s success in identifying specific loads and the accuracy of the energy consumption estimates. A performance assessment protocol was also developed to address common issues with NILM mislabeling and ground-truth comparisons that have not been sufficiently addressed in past evaluations. The NILM product’s estimates were compared to the submetered consumption of eight major appliances. Overall, the product had good performance in disaggregating the energy consumption of the electric water heaters, which included both electric resistance and heat-pump water heaters, but only a fair accuracy with refrigerators, dryers, and air conditioners. The performance was poor for cooking equipment, furnace fans, clothes washers, and dishwashers. Moreover, the product was often unable to detect major loads in homes. Typically, two or more appliances were not detected in a home. At least two dryers, furnace fans, and air conditioners went undetected across the eight homes. On the other hand, the dishwasher was detected in all homes where available or monitored. The key findings were qualitatively compared to those of past field evaluations. Potential areas for improvement in NILM product performance were determined along with areas where complementary technologies may be able to aid in load-disaggregation applications.

## 1. Introduction

Researchers have long sought less-intrusive methods of obtaining end-use energy consumption data. These data are essential for forecasting at an aggregate level and improving energy efficiency at a single-building level. An attractive means of collecting these data is through nonintrusive load monitoring (NILM). NILM is the application of data analytics and techniques to whole-building or aggregate electrical power data to identify the specific end uses operating during each interval and estimate their electrical consumption. 

NILM technologies typically analyze power signatures obtained from measurements taken at a single point in each building (e.g., electrical mains) [1]. Using these measurements, NILM technologies use algorithms and continually evolving data libraries to identify specific end uses and break out their electricity consumption. The NILM approach is compelling because it has the potential to scale across the large population of buildings equipped with smart meters, smart panels, and/or energy management systems.

More NILM products are appearing in the marketplace with the primary objective of disaggregating residential energy consumption. At the same time, innovative appliances and loads are constantly emerging. For these reasons, it is important for prospective users to understand the capabilities of NILM products to disaggregate emerging loads and how the products compare to one another. Pereira and Nunes [2] identified the need to consider real-world environments. Nevertheless, only a few studies have evaluated the field performance of NILM products. This paper’s contributions are as follows:

- A review of key findings from NILM field evaluations;
- A protocol for field evaluations of NILM products;
- A field evaluation of a state-of-the-art NILM product, using recommended metrics by a technical advisory group (TAG);
- A hybrid load-disaggregation approach using NILM and complementary technologies.

Together, these contributions advance the use of NILM products toward more effective methods of load disaggregation. 

## 2. Related Work

Performance evaluations are an integral part of the development of NILM products, algorithms, and technologies. (NILM product refers to a commercially available product or service in the market. NILM algorithm refers to the logic and intelligence applied in the disaggregation of loads. NILM technologies refer to combinations of hardware, software, algorithms, and communications). A “ground truth” is essential for a fair assessment of NILM performance, but there is a wide variation in sources and methods for obtaining the ground truth. Sources of ground truth include public datasets and direct load measurements from actual buildings or laboratory environments. Most NILM algorithms, in the early stages of research and development, have been validated using previously curated public datasets [3,4]. Only a few studies have tested NILM products in occupied homes with data newly captured directly by evaluators [3,4,5,6]. For commercial buildings, only four field evaluations of NILM have been reported [7,8,9,10].

Consistent metrics for evaluating NILM technologies are important for comparing performance results of different studies and gauging the maturation of NILM products. Several different metrics have been proposed and used to assess the success in identification and disaggregation [1,2]. The suitability of many of the NILM performance metrics proposed in the literature was compared and analyzed for an NILM TAG of diverse industry stakeholders (e.g., utilities; regional energy-efficiency organizations; and NILM vendors, researchers, and evaluators) [1]. The TAG agreed that the match rate metric should be used for assessing energy estimation performance in 2016 [1]. However, validation studies of NILM performance have yet to incorporate this metric. Thus, it continues to be difficult to compare NILM systems, even when tested against the same public dataset [4]. Comparisons of NILM systems in actual buildings either have been nonexistent or have not been made publicly available.

The absence of standard protocols and metrics for NILM performance evaluations has prevented quantitative comparisons of results across different field studies [5]. Real-world environments add another level of complexity when assessing NILM performance. Most ground-truth datasets are short, incomplete, and full of errors. This is no surprise because a ground truth is expensive to collect and clean due to budget constraints and unique categorizations of disaggregated energy consumption by NILM vendors, and it can be very challenging to find appropriate matches between NILM outputs and ground-truth data to assess the performance [5]. As a result, NILM performance can be misinterpreted. In [5], many ground-truth data combinations were compared to NILM outputs to assess performance. After relabeling and comparing NILM outputs with the most correlated ground-truth data, the performance in estimating the energy use of some end-use cases significantly improved. However, no other field studies have highlighted the challenges faced during evaluations or how to work around those issues for the fairest assessment of NILM performance. Future evaluations are certain to face similar issues; therefore, an industry standard protocol is warranted so that evaluations can be performed and interpreted consistently.

### Findings from Prior NILM Field Evaluations

Quantitative results of three prior field studies [5,11,12] for evaluating NILM products have been made publicly available in the last decade. Although these studies do not use the same metrics or protocols to validate the performance, the findings may be used to qualitatively assess the capabilities of NILM products. In [11], a smart-meter NILM product was tested on 15 min whole-home energy use data collected from 264 homes over a 12-month period. An average error of 30–45% in monthly energy predictions of four major appliances (HVAC, refrigerator, clothes dryer, and dishwasher) was observed from an NILM product. In [5], a 70% energy accuracy for daily energy consumption of ON/OFF loads (e.g., electric resistance water heaters, clothes dryers, and furnaces) was reported for the most successful of three different NILM products evaluated across 30 homes. The study results were based on 5 min resolution data collected over 6 months and required a manual relabeling of NILM outputs. White and Esser [12] assessed the performance of four NILM products that were provided with whole-building smart-meter inputs. The highest-performing NILM in that study demonstrated energy accuracies ranging from 74 to 88% for electric vehicles, pool pumps, and refrigerators.

The performance varied significantly across homes included in field studies [11], likely due to differences in appliances available in each home. These products were most successful at estimating energy consumption of ON/OFF loads with large power magnitudes and/or regular runtimes such as electric water heaters, clothes dryers, furnaces, electric vehicles, and pool pumps [5,12]. However, these products struggled with all other loads of focus in the NILM evaluations, such as clothes washers, refrigerators, freezers, cooking appliances, and dishwashers. From [11], it was found that NILM products tend to underestimate consumption. This is probably due to their inability to capture standby loads and challenges with classifying individual functions of multistate loads, such as the compressor, icemaker, and defroster in refrigerators [5]. Capturing continuous and standby loads was also identified as a serious shortcoming in a field evaluation of three commercial buildings [7].

Improved performance was exhibited when higher resolution data were used as inputs for NILM products and when given a priori information about appliances in each home [5,12]. The study reported that the best results were derived from 10 s resolution data inputs compared to 1 min, 15 min and 1 h data. NILM products had improved performance when NILM outputs were relabeled according to ground-truth data that were more correlated in [5]. NILM in [12] had improved performance once information from home appliance surveys was provided as inputs for disaggregation.

## 3. Methodology

To address the lack of evaluation of state-of-the-art NILM products, a field evaluation was conducted to assess the disaggregation performance of several NILM products. The NILM products were selected based on responses by vendors to a questionnaire about their commercially available products. Single-family homes were then selected using a convenience sampling approach. The NILM products and ground-truth submeters were installed in the homes, and a subset of residential end-use categories was chosen as the focus of the NILM performance evaluation. 

The field evaluation was split into two phases. In the first phase, data were collected for a year in each home. At the end of the year, the performance of the most promising products was compared. The product with the superior performance at the end of the first phase was considered representative of the state-of-the-art NILM technology and was selected to continue to the second phase of the evaluation. In the second phase, the representative product was installed in all homes, and data were collected for another year. The overall performance of the NILM selected for the second phase was assessed based on data collected in both phases. The performance assessment metrics and protocols used are discussed in the following subsections. The key findings from this field study as well as prior NILM field evaluations are then examined to recommend a direction for future research and hybrid load-disaggregation approaches. 

### 3.1. Field Evaluation Performance Assessment Metrics

The following metrics were used to compare the performance of NILM products for the residential evaluation: match rate (*MR*), mean bias error (*MBE*), and end-use detection. 

The *MR* metric was recommended by an NILM TAG, as described in [1], for the NILM energy estimation performance of individual loads or end uses [2]. This field evaluation is the first application of the *MR* metric and is the first step toward an industry consensus on the use of this metric. The *MR* is defined in Equation (1):

(1) $$MR=\frac{\sum_{i=1}^{N}\min\{E_{i},\hat{E_{i}}\}}{\sum_{i=1}^{N}\max\{E_{i},\hat{E_{i}}\}}\times 100\%,$$

where $\hat{E_{i}}$ = estimated energy usage (NILM disaggregated) of a specific load at each time interval *i*, $E_{i}$ = ground-truth energy usage (metered) of the corresponding load at each time interval *i*, and *N* is the number of intervals over a time series. 

**Figure 1:**
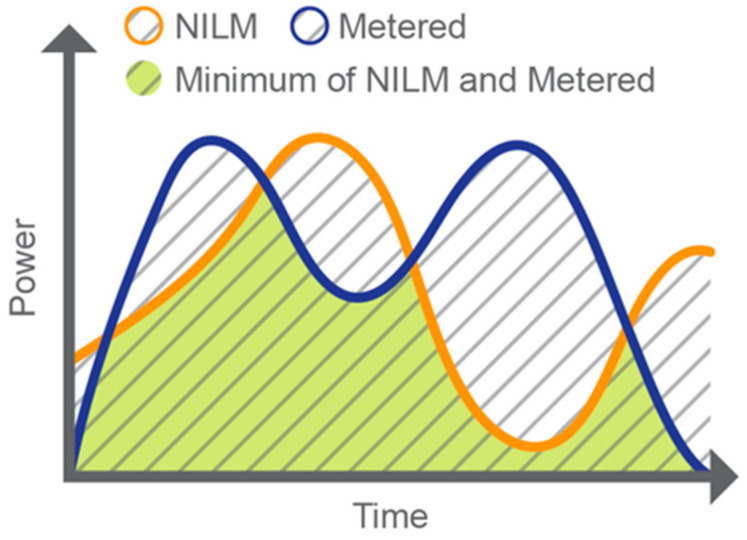
Match rate metric illustration, where the numerator is shaded in yellow with gray stripes and the denominator is the entire area with gray stripes.

The *MR* metric is suitable for time series data and can consider an error due to the over/underestimation of missed and/or falsely identified events and offsets in data. Figure 1 illustrates how the *MR* metric is calculated for each individual load. It compares the NILM estimated (disaggregated) values to the metered ground-truth (actual) values at each time interval to find the minimum and maximum values, then sums these values over the time series. In Figure 1, the numerator is shaded in yellow with gray stripes, and the denominator is the entire area with gray stripes. The numerator is always less than or equal to the denominator. Therefore, the metric is bound between 0% and 100% (the match rate may be greater than 100% or less than 0% with few exceptions, such as when disaggregation accounts for solar photovoltaics generation, battery storage, measurement error, and other considerations). The metric value will be 100% only when the two time series under comparison are a perfect match. The *MR* was appropriate for this study since the objective was to understand accuracy. Three performance levels were defined: good (*MR* ≥ 70%), fair (50% ≤ *MR* < 70%), and poor (*MR* < 50%). 

*MBE* is used to understand whether a disaggregation technology tends to overestimate or underestimate the energy consumption of different end uses. For this evaluation, *MBE* was computed using Equation (2):

(2) $$MBE=\frac{1}{N}\sum_{i=1}^{N}\left({\hat{E}}_{i}-E_{i}\right),$$

where ${\hat{E}}_{i}\;$= estimated energy usage (disaggregated) at each time interval *i*, $E_{i}\;$= ground-truth energy usage (metered) at each time interval *i*, and *N* is the number of intervals over the time series.

### 3.2. Performance Assessment Protocol

A consistent procedure to match NILM outputs to ground truth is a fundamental requirement for evaluations. The details of the protocol used in this study are described in Appendix A. Some practical problems of applying this matching protocol are discussed below. 

**Figure 2:**
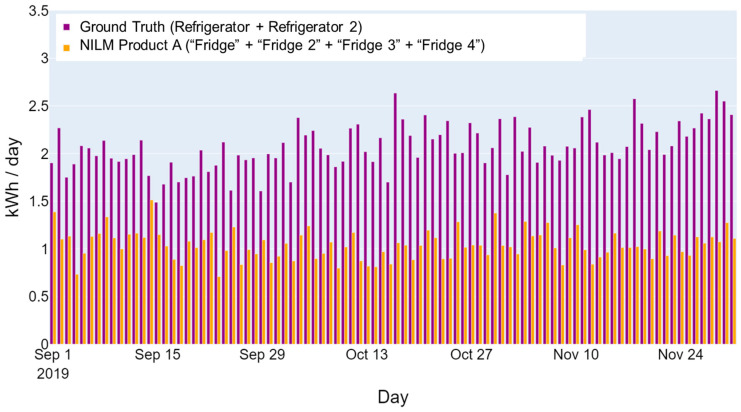
Example of a home refrigerator’s daily energy consumption—includes two monitored refrigerator loads in the home.

**Figure 3:**
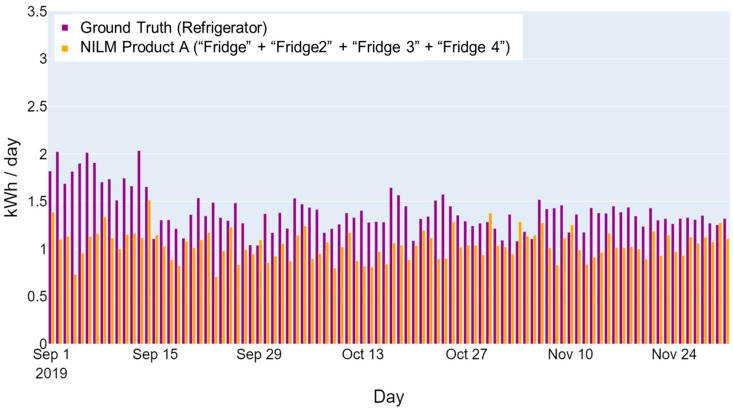
Example of a home refrigerator’s daily energy consumption—includes only one monitored refrigerator load in the home.

The lack of one-to-one matches between the NILM outputs and ground truth is the primary difficulty for a performance evaluation. For example, there may be two refrigerators present in a home (identified by ground-truth labels “Refrigerator” and “Refrigerator 2”). However, an NILM product may identify multiple refrigerators or components, (e.g., “Fridge”, “Fridge 2”, “Fridge 3”, and “Fridge 4”). The question becomes, what data should be used to assess the product’s ability to disaggregate refrigerators? Figure 2 and Figure 3 show how different data combinations may provide a better alignment between NILM outputs and ground truth. The bar graphs show the daily energy consumption of the ground truth (displayed in purple) and NILM outputs (displayed in orange) from an actual home. As shown in Figure 2, the total disaggregated consumption by the NILM product (“Fridge” + “Fridge 2” + “Fridge 3” + “Fridge 4”) is much lower than the total consumption of both refrigerators monitored (“Refrigerator” + “Refrigerator 2”). However, when only the “Refrigerator” ground truth is compared to the total disaggregated refrigerator consumption (shown in Figure 3), the disaggregated refrigerator energy use aligns better with the ground truth. 

**Figure 4:**
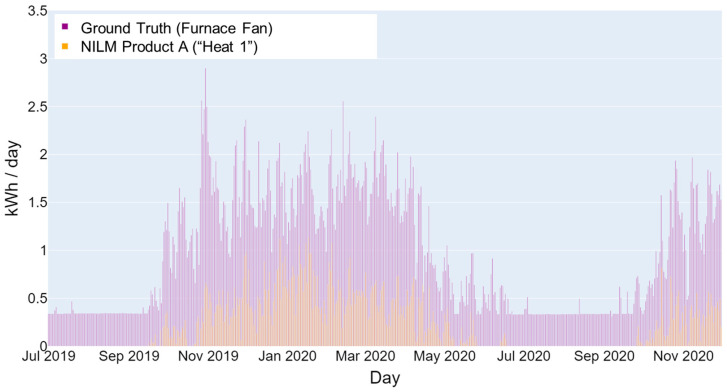
Example of a home furnace fan’s daily energy consumption.

In other cases, NILM products may identify and categorize energy consumption (e.g., heat 1, heat 2, etc.) that does not directly correspond to specific end uses monitored for ground truth (e.g., furnace fan, kitchen oven range, water heater). This makes it difficult to determine which data to compare for some end-use categories. This ambiguity also exists for broader NILM energy-use categories (e.g., cooking, heat, always on) that do not correspond to a single end use. Figure 4 further illustrates this point, where the “Heat 1”-labeled energy consumption (displayed in orange) by an NILM product appears to be an attempt to disaggregate the energy consumption of a furnace or other types of heating components (displayed in purple). The “Heat 1” energy consumption seems to follow the actual energy use trends of the furnace fan, even though the energy use was significantly underestimated, by approximately 75% (shown in Figure 4). A significant reason for the underestimation is likely that the NILM product failed to capture the standby power consumption of the furnace fan controls (most evident in months of July through September), which the NILM product may have assigned to the “Always On” category.

A similar kind of mismatch between NILM and ground-truth labeling is where the NILM identifies the energy consumption of a ductless heat pump (DHP) but mislabels it as a motor. As a result, the accuracy in disaggregating DHP energy use might be determined to be 0% and/or “not detected” in this case. However, if the motor data labeled by the NILM product were compared to the DHP ground truth, the accuracy for the DHP would be 59%, which corresponds to a “fair” performance accuracy. These mislabeling issues are broadly consistent with those reported by McWilliams et al. [6].

**Figure 5:**
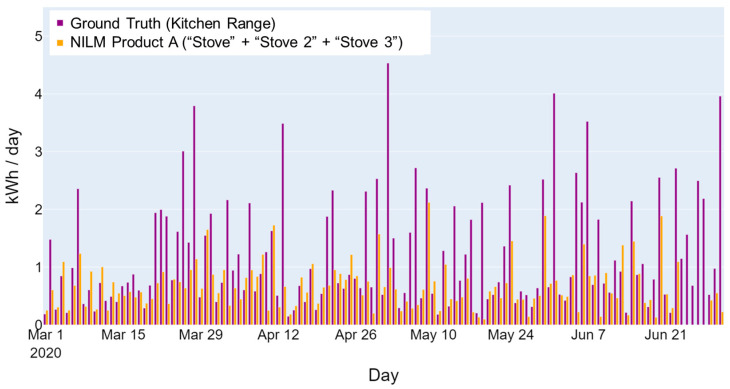
Example of home cooking appliances’ daily energy consumption.

The NILM cooking category, which may include ranges, stovetops, and ovens, is another example of an NILM output that is susceptible to misinterpretation. Some homes may include dual fuel ranges, a stovetop that is separate from ovens with different fuel types or a combined electric oven and stove (referred to as a range). The ground truth in different homes may include a stove, oven, or both, and NILM products may detect each separately. Figure 5 further illustrates this example, showing the ground-truth energy consumption for an all-electric range (“Kitchen Range”, displayed in purple) monitored in a home. The NILM product appears to only detect stove energy use and not the oven, as indicated by its energy consumption labels (“Stove” + “Stove 2” + “Stove 3”, displayed in orange). The question becomes how to fairly assess the performance of the cooking category. From a visual inspection, the stove consumption disaggregated by the NILM product tracks the kitchen range energy use. However, because the NILM product is unable to also detect oven use, the accuracy based on the *MR* metric is computed to be 42%. In this case, the ground truth for the kitchen range cannot be separated to evaluate the NILM’s ability to disaggregate the energy consumption of the stove only. 

In summary, the issues with one-to-one matches in ground truth and NILM outputs can be due to limitations of either the ground truth captured or the NILM product labeling. 

## 4. Experimental Design and Setup

For the field evaluation of NILM products, the research team initially evaluated eight residential NILM products in terms of the following attributes: the installation and configuration process, maintenance support, hardware costs, and communication strength and requirements. Three products with unique features were selected. The assessment involved determining the measurement accuracy of load disaggregation products using submetered data as the baseline and then comparing the measurement accuracy among products to the greatest extent possible using the *MR* and *MBE* metrics. The NILM products were evaluated based on their ability to measure the total electricity for the home, detect end uses, measure/estimate the electricity consumption of end uses, and perform reliably without burdening participants. 

### 4.1. Evaluation Design

The goal of the field evaluation was to assess the performance of NILM products. The best candidates were selected through a screening process. First, a questionnaire was sent to 12 vendors. The questionnaire covered hardware costs, installation costs/time, communication requirements, data storage and buffers, and the ability to access disaggregated time interval power/energy data. From the eight vendors that responded, three products stood out because of their unique technologies and perceived potential to provide more accurate energy estimates; these are referred to as products A, B, and C. The second step in the selection of a state-of-the-art product involved a pilot project for products A, B, and C. The second year involved the collection of more data on the products that showed the most promise based on preliminary results obtained 1 year into the study [13].

Eight end uses were chosen as the focus of the NILM performance evaluations: refrigerator, clothes dryer, dishwasher, cooking, furnace fan, air conditioner, water heater, and clothes washer. These loads were selected in part because they are relatively large and are typically connected to dedicated circuits. The dedicated circuit requirement greatly simplified submetering because the sensors could be attached at the electric breaker panel rather than at the device. 

All of the evaluated homes were located near Portland, Oregon, USA, which is a mixed marine climate (International Energy Conservation Code climate zone 4C). A screening process was also used to select the homes to participate in the field evaluation. Candidate homes were scored based on the number of major electric end uses and the anticipated complexity of installation. The eight highest-rated candidates were invited to participate in the study. The floor areas of the homes ranged from 195 to 325 m^2^. Data from NILM products were captured for approximately 2.5 years (June 2018–December 2020).

### 4.2. Installation, Configuration, and Maintenance

Each NILM product chosen had a unique product design. Product A consisted of a set of two current sensors installed on the electrical mains at the panel, and connected to a processor that was small enough to fit within the panel. The sensors measured both current and voltage, sampling measurements at a 1 MHz interval for disaggregation, which was appealing since the high sampling rate could lead to a higher accuracy. Product A used a gateway to transmit disaggregated estimates to the cloud via Wi-Fi. 

Product B used an optical sensor to read data from the utility meter. An infrared sensor system was attached to the utility meter to read meter pulses and obtain power inputs, sampling every 15 s. Data were communicated to the cloud via Wi-Fi or Ethernet at a bandwidth of less than 25 MB/day. This design dramatically reduced the cost and complexity of installing the product, making it possible for homeowners to install it. Therefore, the deployment potential for this product appeared to be much higher than other designs.

Product C had a combination of direct submetering and NILM analytics to disaggregate loads. Four individual circuits are submetered by the product’s current sensors to capture major or important loads on dedicated circuits, and the remaining electrical load is disaggregated by a processor. It communicates measurements via Wi-Fi with a low bandwidth of less than 10 MB/day. This product was larger and more complicated to install than products A and B. 

**Figure 6:**
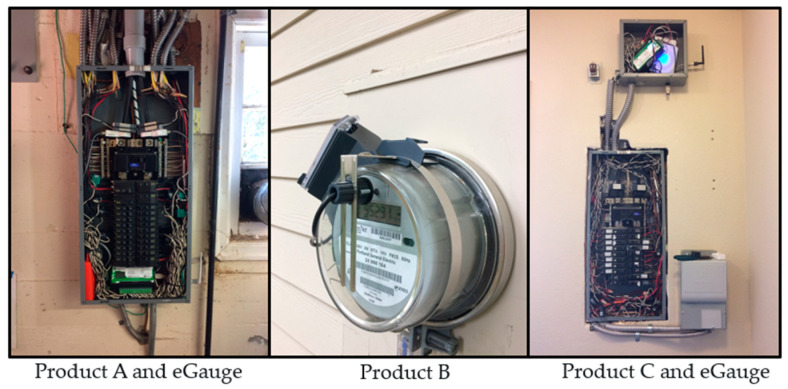
From left to right, product A and eGauge submeter installed in the panel, utility meter with product B installed, and product C and eGauge installed in an enclosure beside the panel.

The ground truth was collected with a 15-channel eGauge submeter with current transformers connected at the electrical panel. The ground-truth submeter and products A and C required connections at the electrical panel; only one of those NILM products could be installed in a home at a time. Therefore, products A and C were initially installed in four homes each, whereas product B was installed in all eight homes. Figure 6 provides details of the connections for the eGauge and products A, B, and C.

The first year of the study encountered product malfunctions and communications disruptions. Maintaining the network connectivity of the submeter and NILM products was an ongoing effort even with dedicated modems and Wi-Fi repeaters. The submeter and NILM products were located in garages, basements, and utility meters with multiple walls (including concrete walls) separating them from the network equipment (i.e., dedicated router or proprietary receiver). Initial testing of Wi-Fi strength in each home identified enclosures as hindering connectivity. USB extension cables were installed on the submeter and product C to bring Wi-Fi receivers out of enclosures and strengthen communications. When the submeter and/or NILM products went offline, the devices or home network equipment were rebooted. These methods resolved connectivity in most, if not all, instances of offline equipment. Overall, the installation and operation of the selected NILM products were much more difficult and less reliable than expected. These problems resulted in incomplete data for all the NILM products evaluated. Even the state-of-the-art product A had approximately 8% of total data loss over 18 months. 

Product C provided energy consumption estimates of end uses through its dashboard, but its 5 min time interval data was unavailable through the API (this was not apparent from the product description). These communication, data accessibility, and consumer engagement problems persisted, so all product C units were removed from the study after 4 months. 

Product B was able to detect only six of the eight priority end uses. This product demonstrated poor performance for all six loads based on the *MR* metric. While product B had promising characteristics to scale up load disaggregation, it was excluded from evaluation in the second phase of the study due to its accuracy and communications limitations. Based on the *MR* results of the first phase, the second phase was refocused on product A, considered the state-of-the-art. The performance evaluation from the first phase is discussed in detail in [13]. 

## 5. Results

**Figure 7:**
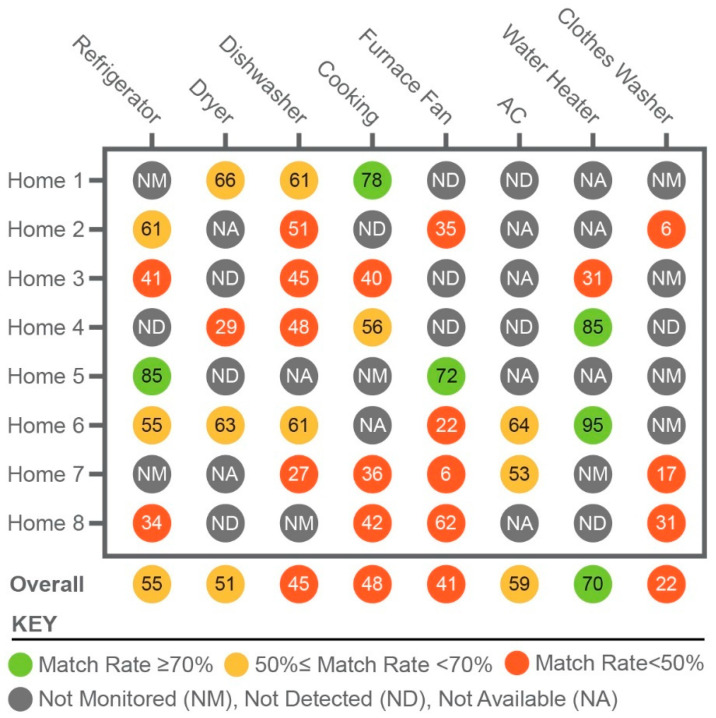
Disaggregation performance of NILM product A.

The *MR* and *MBE* metrics described in Section 3.1 were used to compare NILM product outputs to ground truth to evaluate performance. Figure 7 summarizes the *MR* results. The green, yellow, and orange cells indicate a good (*MR* ≥ 70%), fair (50% ≤ *MR* < 70%), and poor (*MR* < 50%) performance accuracy, respectively.

Gray cells indicate that the end use in the corresponding home was either not monitored (NM), not available (NA), or not detected (ND) by the disaggregation product, and therefore was excluded from the assessment. The ground truth was insufficient in some cases to determine whether the NILM products were identifying end uses accurately. At the same time, an NILM product may have identified certain end uses that were not monitored in the ground truth. Thus, the NILM performance was not evaluated for cases where either the ground truth (specified as NM or NA) or estimated energy consumption for end uses was not available (specified as ND). For example, in home 1, product A demonstrated a good performance in disaggregating the cooking end uses and a fair performance for the dryer and dishwasher. The results for the water heater, as indicated by the gray cell with NA, were excluded since the water heater was gas-fueled instead of electric. Each product’s overall performance in disaggregating each priority end use is also provided in Figure 7, which was determined based on the weighted average of the *MR* across all homes.

According to the rating scale, product A had good overall performance in disaggregating the energy consumption of the electric water heaters, which included both electric-resistance and heat-pump water heaters. Home 5 had a heat-pump water heater, and product A’s accuracy in disaggregating the usage was well above the average for all homes. A fair rating was given for disaggregating the energy use of refrigerators, dryers, and air conditioners. The performance was poor for the cooking equipment, furnace fan, clothes washer, and dishwasher. 

Product A was often unable to detect major loads in homes. Typically, two or more appliances were not detected in a home. At least two dryers, furnace fans, and air conditioners went undetected across the eight homes. On the other hand, the dishwasher was detected in all homes where available or monitored. 

Table 1 summarizes the *MBE*s of product A for each end use. For all but three end uses detected, product A generally underestimated the energy usage. 

## 6. Discussion

The chief applications of load disaggregation data require different levels of performance accuracy, which should be determined by users of the data. Regardless of the application, the first step is to understand the NILM’s performance through an evaluation. Only then can users decide if an NILM product is adequate for their applications. The following discussion is divided into insights from the field evaluation presented in this paper and prior field studies. These insights are then used to make recommendations for the direction of future research.

### 6.1. Key Findings 

The findings of the field study presented in this paper and prior studies highlight the need for the following: (1) a labeling standard for loads in buildings, (2) the consistent use of performance assessment metrics and protocols, and (3) a narrower focus of NILM on disaggregation loads that may be captured in real-world environments. 

The absence of an NILM industry classification or labeling standard causes confusion in NILM performance assessments and misinterpreted results. Since NILM vendors uniquely label electric loads and the same ground-truth data cannot be collected from each home, selecting the appropriate ground-truth comparisons for disaggregated outputs is not a straightforward process. A labeling standard for building loads could remove issues that arise when reconciling disaggregated outputs (e.g., whether the energy consumption of both the stove and oven is captured in the cooking category, what is meant by a motor 1 label).

Consistent metrics and protocols are the key elements to credible and comparable evaluations. They are also useful for tracking technology maturity over time. The field study in this paper is the first to apply the *MR* metric that was recommended by an NILM TAG in 2016. The results demonstrate that the metric is useful for determining the accuracy of disaggregated energy consumption. The application of the metric is also a step toward an industry consensus on its use. The *MBE* metric was also used to understand under- and overestimation tendencies. This metric is also recommended for future evaluations. 

Past studies have not sufficiently addressed issues with ground truth and NILM output comparison, which can seriously undermine evaluations. The performance assessment protocol developed for this study helps to address these issues and leads to fairer performance assessments. This protocol is proposed for future NILM evaluations and validation studies as it is effective in sorting, matching, and maximizing the use of the data collected for NILM evaluations. It allows for evaluators to move past some of NILM mislabeling issues to glean deeper insights. However, the protocol has limitations. It is unable to split ground-truth data into individual components for comparison to individual loads (e.g., oven or stove) disaggregated by NILM technologies. For example, the ground truth for a cooking range may not be split into the individual oven and stove components for comparison to the oven disaggregated energy usage. In these cases, the NILM performance for an oven may be misinterpreted because the outputs may be compared to a cooking range’s ground truth. The protocol also does not specify which loads should be the focus of the NILM evaluations or timeframes for evaluations, such as training and assessment periods. The real-world studies examined demonstrated that NILM technologies need significant improvement if policymakers, building occupants, operators, and utility planners are to rely on them. 

So far, NILM products have demonstrated a potential to estimate the energy consumption of relatively large and cyclic legacy loads in residential applications, such as refrigerators, water heaters, pool pumps, electric vehicles, furnaces, clothes dryers, and ovens. However, multistate loads present challenges with grouping individual functions, such as compressors, icemakers, and defrosters, into refrigerator categories. In addition, there is a significant variance in accuracy across diverse homes and loads. Increased data resolution has been shown to improve accuracy. On the other hand, NILM products have exhibited insufficient capabilities to decipher smaller devices (e.g., plug and stand-by loads) and a tendency to underestimate loads. These highlighted characteristics signal the need for high-resolution data sampling (subsecond) for NILM product inputs, complementary technologies that may provide certainty on the operation of loads, as well as more intelligent algorithms such as the artificial intelligence approach presented in [14]. Future research should also focus on improving detection and energy estimation with a focus on larger cyclic and ON/OFF loads commonly available in current homes.

### 6.2. Broader Implications 

Moving forward, novel ways of measuring, estimating, and reporting end-use energy consumption are needed. NILM appears to be more useful as a bridging or complementary technology to those new methods of collecting disaggregated end-use data rather than operating in isolation. The following technologies should be considered going forward to develop more reliable hybrid load-disaggregation approaches.

One promising new method is self-reporting. Self-reporting end uses have the measurement, estimation, and reporting technology embedded within the device. This approach is advantageous for collecting data. First, communication capabilities exist in many products (e.g., Internet of things (IoT) devices). Second, self-reporting resolves the issue of device identification, as each individual device can report its make, model, relevant specifications, and status. Third, self-reporting does not require direct metering of electricity use when proxies can provide acceptable accuracy. The simplest energy-use proxies are a sum product of the average power drawn by mode with the elapsed time in each mode. 

In the past, self-reporting was challenged by the lack of interoperability and communication between devices with proprietary protocols. The recent finalization of the Connectivity Standards Alliance’s Matter open standard should help resolve this issue moving forward. To date, the accuracy of self-reporting devices has yet to be determined due to a lack of research. However, by embedding metering hardware into devices or using proxies, the accuracy has the potential to be similar to plug load monitors or circuit-level submeters.

Similar to self-reporting, the latest USB-PD 3.1 standard expands opportunities for USB-C to supply power to an increasing number of devices (up to 240 W in power), and thus provide reporting on the device (e.g., identification) and its operation (e.g., battery charge levels, voltage, and current). Currently, retrieving power information through USB-PD is a challenge. However, low-power wireless communication, such as Bluetooth mesh, may offer a solution, particularly with the Matter open standard providing integration and interoperability for IoT devices and apps. 

Over the long term, self-reporting devices and USB-PD can provide valuable information that can improve device power scaling, power management, and usage. However, since these technologies require a network connection, a different technology is needed to collect energy use data for the many unconnected devices in the market and building stock. While NILM technologies have not performed with the same level of accuracy as direct submetering technologies, there is the potential for the interoperation of self-reporting and USB-PD with NILM to improve end-use identification and energy use estimation. If self-reporting and USB-PD were interoperable with NILM, the number of devices for NILM to identify would be reduced from the aggregate, which could simplify the device identification and estimation process for NILM. 

The latest smart-panel technologies are another conduit for NILM to collect energy use data for unconnected devices. The demand for building electrification, electric vehicles, and distributed energy resources (e.g., solar photovoltaics) has required that many homes upgrade their electrical panels. New smart panel technologies are equipped with submetering at the circuit level. This allows for the direct measurement of end uses on dedicated circuits. It also provides an opportunity to integrate NILM into smart panels to disaggregate shared circuits individually. The disaggregation of end uses at the circuit level reduces the number of devices that the NILM needs to identify and estimate. If the smart panel and NILM were also interoperable with self-reported devices and USB-PD, the disaggregation of end uses could be simplified further, potentially improving NILM identification and estimation. 

Measurement, estimation, and reporting technologies vary in their technological advancements, market adoption rates, and consumer preferences. Any given building may have a combination of self-reporting devices, NILM, smart panels, plug load monitors, and USB that can perform energy-use monitoring, collection, and reporting. The interoperability of these technologies can improve the accuracy and comprehensiveness of the device monitoring and collection process. When technologies work in concert, they can provide a greater context and fill any data gaps. To do so, they will need a way to network and communicate, perhaps through a centralized hub with multiple communications capabilities (e.g., Wi-Fi, Zigbee, USB-C, Power over Ethernet).

## 7. Conclusions

This paper presented results from a field evaluation of the performance of a state-of-the-art NILM product. The key findings were then compared to those of past field evaluations. Potential areas for improvements in NILM product performance were determined along with areas where complementary technologies may aid in load disaggregation applications. 

Only a few NILM algorithms have successfully transitioned to commercial applications, and field evaluations and publications of their results are rare and limited. Three advancements are still needed to enable NILM to become a widely useful technology: (1) a labeling standard for loads in buildings, (2) the consistent use of performance assessment metrics and protocols, and (3) a narrower focus of NILM on loads that can be captured in real-world environments.

The field study presented in this paper found that *MR* was an effective metric due to its ease of application and clarity of interpreting its results. In particular, *MR* provides insights because it accounts for both under- and overestimation in its assessment of NILM performance. The *MBE* was also useful for understanding whether NILM products tend to under- or overestimate energy consumption. A performance assessment protocol was also developed to address common NILM mislabeling and ground-truth comparison issues and to enable evaluators to glean deeper insights. The protocol was effective in sorting, matching, and maximizing the utilization of the data collected for NILM evaluations. Future development efforts should address the capture of ground truth that can compare directly to NILM outputs, specific loads, and timeframes. Future NILM evaluators are encouraged to adopt the same metrics and protocols so that future studies can be quantitatively compared.

Despite enormous improvements in algorithms, metering equipment, and computation power, the NILM approach has not successfully demonstrated confidence and reliability for widespread adoption. Currently, no single technology provides an accurate, comprehensive, and economical solution to understanding disaggregated building energy consumption. Therefore, future work should pair NILM with other promising data-collection technologies, such as device self-reporting and circuit-level sensors, which may yield more accurate results at a low cost. Regardless of the improvements, field evaluations in realistic environments will be an essential step in validating NILM and giving it legitimacy.

## Figures and Tables

**Table 1:** Mean bias errors of NILM product A.

Mean Bias Error	Home ID	Refrigerator (kWh)	Dryer (kWh)	Dishwasher (kWh)	Cooking (kWh)	Furnace Fan (kWh)	AC (kWh)	Water Heater (kWh)	Clothes Washer (kWh)
Product A	1	NM	0.38	0.05	−0.16	ND	ND	NA	NM
	2	−0.60	NA	−0.34	ND	−0.69	NA	NA	−0.22
	3	−0.77	ND	−0.21	−0.49	ND	NA	−3.50	NM
	4	ND	−1.20	−0.36	−0.52	ND	ND	−0.20	ND
	5	0.12	ND	NA	NM	−0.19	NA	NA	NM
	6	−0.22	−0.37	−0.13	NA	−1.78	−0.09	−0.52	NM
	7	NM	NA	−0.46	−0.70	−3.03	−0.21	NM	−0.05
	8	−2.18	ND	NM	−0.49	−0.26	NA	ND	−0.03
	Overall	−0.76	−0.67	−0.23	−0.48	−1.25	−0.14	−1.41	−0.07

## Data Availability

Restrictions apply to the availability of these data. The data are not publicly available due to confidentiality agreements.

## Appendix A

The following protocol was developed to work around the challenges so that NILM products can more fairly be evaluated:

1. Select the end-use categories of interest for evaluating the NILM product. These end uses should be monitored directly to collect ground-truth power data for comparison with NILM outputs.
2. From all disaggregated loads of each NILM in each home, determine all possible combinations up to *m* disaggregated loads. For evaluation purposes, *m* = 3 is recommended. For example, if an NILM product provides three disaggregated outputs labeled “Stove/Range”, “Load 2”, and “Load 3”, as shown in Table A1, the seven possible combinations for up to three loads are “Stove/Range”, “Load 2”, “Load 3”, “Stove/Range” + “Load 2”, “Stove/Range” + “Load 3”, “Load 2” + “Load 3”, and “Stove/Range” + “Load 2” + “Load 3”.

**Table A1:** Sample NILM product output.

Time	Stove/ Range (kWh)	Load 2 (kWh)	Load 3 (kWh)
19 October 2016 0:00	2.888	0.306	0.228
19 October 2016 0:01	4.641	0.306	0.228
19 October 2016 0:02	5.258	0.306	0.228
19 October 2016 0:03	5.258	0.306	0.228
19 October 2016 0:04	5.258	0.306	0.228
19 October 2016 0:05	5.258	0.306	0.228
⋮	⋮	⋮	⋮

3. Sum the NILM energy estimates of the loads within each disaggregated load combination, determined in step 2, to get the total power at each time interval. Table A2 provides an example.

**Table A2:** All possible combinations of disaggregated outputs for the example NILM product.

Time	Stove/Range (kWh)	Load 2 (kWh)	Load 3 (kWh)	Stove/Range + Load 2 (kWh)	Stove/Range + Load 3 (kWh)	Load 2 + Load 3 (kWh)	Stove/Range + Load 2 + Load 3 (kWh)
19 October 2016 0:00	2.888	0.306	0.228	3.186	3.116	0.534	3.414
19 October 2016 0:01	4.641	0.306	0.228	4.947	4.869	0.534	5.175
19 October 2016 0:02	5.258	0.306	0.228	5.564	5.486	0.534	5.792
19 October 2016 0:03	5.258	0.306	0.228	5.564	5.486	0.534	5.792
19 October 2016 0:04	5.258	0.306	0.228	5.564	5.486	0.534	5.792
19 October 2016 0:05	5.258	0.306	0.228	5.564	5.486	0.534	5.792
⋮	⋮	⋮	⋮	⋮	⋮	⋮	⋮

4. Identify which disaggregated load combinations demonstrate the best fit with the ground-truth data for each end-use category. Find the closest NILM output match for each end-use category by comparing the ground truth collected for each category to each disaggregated load combination determined in step 3. The *r*^2^ metric below can be used for this comparison to examine the “goodness in fit” between power profiles.

(A1) $$r^{2}=1-\sum_{i=1}^{N}\frac{{\left(E_{i}-{\hat{E}}_{i}\right)}^{2}}{{\left(E_{i}-\bar{E}\right)}^{2}}$$

where $E_{i}$ is the observed value, represented by the submetered or ground-truth value for a particular end use, at the *i*th time interval. ${\hat{E}}_{i}$ represents its corresponding predicted value, represented by the disaggregated output, at the *i*th time interval. $\bar{E}$ is the mean of the set of observed values over the test period. *N* is the number of time intervals included in the test period. To use Equation (A1), the units and time resolution of the submetered and NILM output data must be the same. Therefore, the appropriate conversions to the data must first be performed as needed.

The best ground-truth and disaggregated load data matches can be identified by creating a matrix, such as the one provided in Table A3, where each entry represents the $r^{2}$ value for the corresponding end-use ground-truth data and disaggregated outputs. Choose the corresponding disaggregated load combination and ground-truth profiles that result in the highest $r^{2}$ value, above zero, in each row to be used for the NILM performance assessment. If a negative value for the highest $r^{2}$ value is obtained for an end-use category, this is an indicator that the NILM product has not sufficiently detected the end use. Therefore, the end use should be reported as “not detected”. Furthermore, check the data if the same disaggregated load combinations result in the highest $r^{2}$ value for more than one ground-truth category.

**Table A3:** Example *r*^2^ matrix for the comparison of ground-truth data and disaggregated outputs.

$r^{2}$	Stove/ Range	Load 2	Load 3	Stove/Range + Load 2	Stove/Range + Load 3	Load 2 + Load 3	Stove/Range + Load 2 + Load 3
**Refrigerator/Freezer**							
**Clothes washer**							
**Clothes dryer**							
**Dishwasher**							
**Water heater**							
**Central air conditioner/Heat pump**							
**Cooking range**							
**Lighting**							

5. For each best match in ground truth and NILM disaggregated load combinations (determined in step 4), roll up the energy data into the daily energy consumption. Then, apply the *MR* and *MBE* performance metrics.
6. Repeat steps 1–5 for each NILM product assessment in each home.
7. Summarize the overall performance across homes using the weighted averages of each metric based on the data available.
